# Supplementary material for: Microfluidics-Enabled Simultaneous Imaging of Neural Activity and Behavior in Chemically Stimulated, Head-Fixed C. elegans
Source: bioRxiv. 2025 Nov 4:2025.11.03.686171. Preprint. [Version 1] doi: 10.1101/2025.11.03.686171 (PMC12637446; doi:10.1101/2025.11.03.686171)
Supplement: 1 [file NIHPP2025.11.03.686171v1-supplement-1.pdf]

bioRxiv preprint doi: https://doi.org/10.1101/2025.11.03.686171; this version posted November 4, 2025. The copyright holder for this preprint (which was not certified by peer review) is the author/funder, who has granted bioRxiv a license to display the preprint in perpetuity. It is made available under aCC-BY 4.0 International license.

# Supporting Information

## Microfluidics-Enabled Simultaneous Imaging of Neural Activity and Behavior in Chemically Stimulated, Head-Fixed *C. elegans*

Hyun Jee Lee, Julia Vallier, Hang Lu*

School of Chemical & Biomolecular Engineering, Georgia Institute of Technology, Atlanta, GA, USA

*Correspondence: hang.lu@gatech.edu

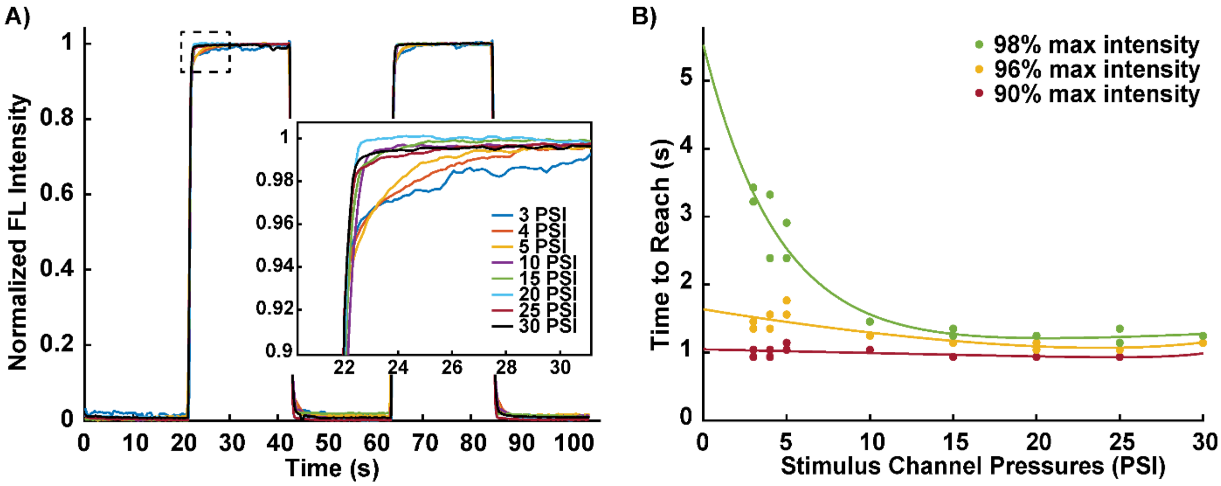


Figure S1: Temporal resolution of chemical stimulation while maintaining head fixation. A) Normalized fluorescence intensity profiles measured near the T-junction during two cycles of fluorescent dye flow at varying stimulus channel pressures. B) Time required to reach 90%, 96%, and 98% of the steady-state (maximum) fluorescence intensity as a function of stimulus channel pressure. Fluid composition switches achieving 90% of steady-state fluorescence or higher can be completed within a few seconds. Solid lines represent exponential fits to the data.

bioRxiv preprint doi: https://doi.org/10.1101/2025.11.03.686171; this version posted November 4, 2025. The copyright holder for this preprint (which was not certified by peer review) is the author/funder, who has granted bioRxiv a license to display the preprint in perpetuity. It is made available under aCC-BY 4.0 International license.

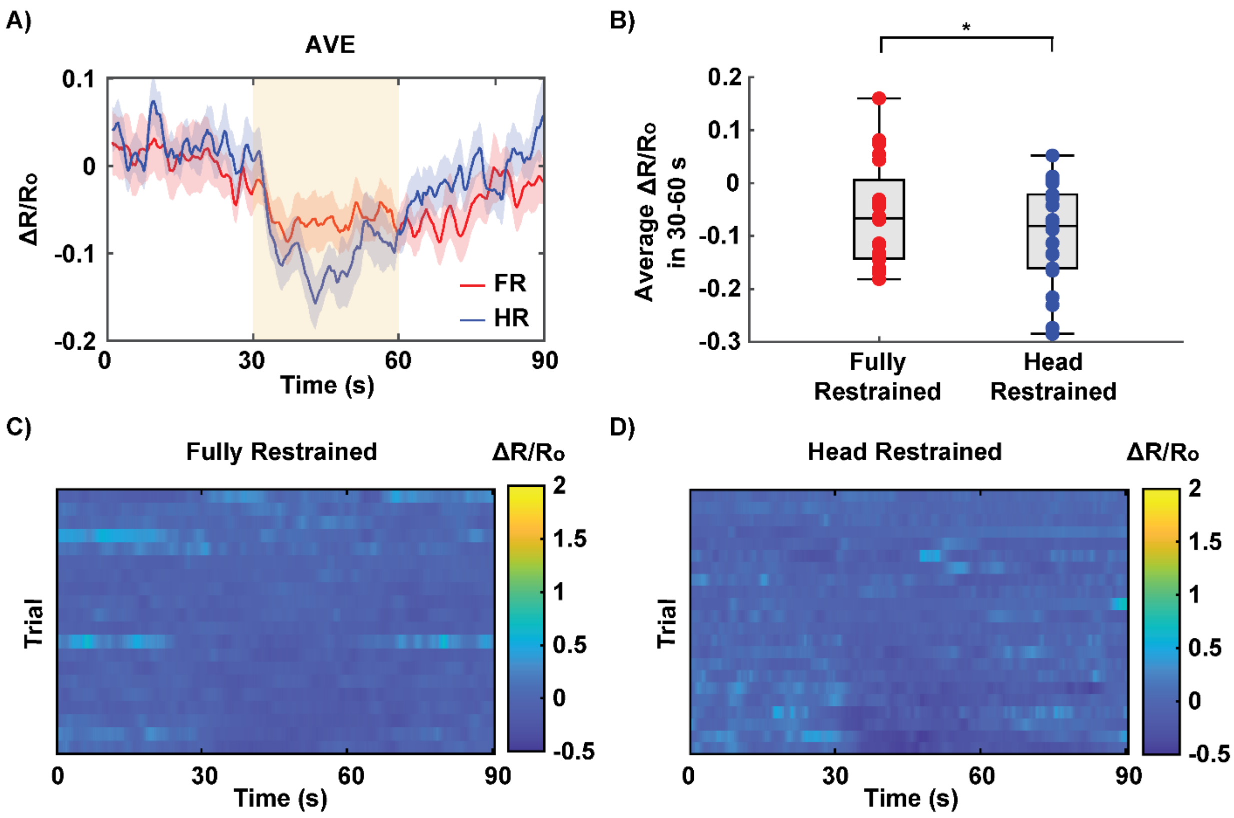


Figure S2: Difference in AVE neuron response in fully-restrained vs head-fixed animals. A) Trial-averaged AVE activity traces (ΔR/R0) of AVE in fully-restrained (FR) and head-fixed (HR) worms. B) Comparison of the average ΔR/R0 values between fully-restrained and head-fixed animals in the 30-60 window. Each dot represents an individual stimulation trial. The box plot displays the median along with the lower and upper quartiles. Statistical significance was determined using two-sample t-tests, with asterisks denoting significance levels: *P < 0.05, **P < 0.01, ***P < 0.001. C,D) Heatmap showing AVE neuron activity (ΔR/R0) across individual trials for fully restrained and head-fixed worms. Each row represents one trial, with the stimulus applied in the 30–60 second window.

bioRxiv preprint doi: https://doi.org/10.1101/2025.11.03.686171; this version posted November 4, 2025. The copyright holder for this preprint (which was not certified by peer review) is the author/funder, who has granted bioRxiv a license to display the preprint in perpetuity. It is made available under aCC-BY 4.0 International license.

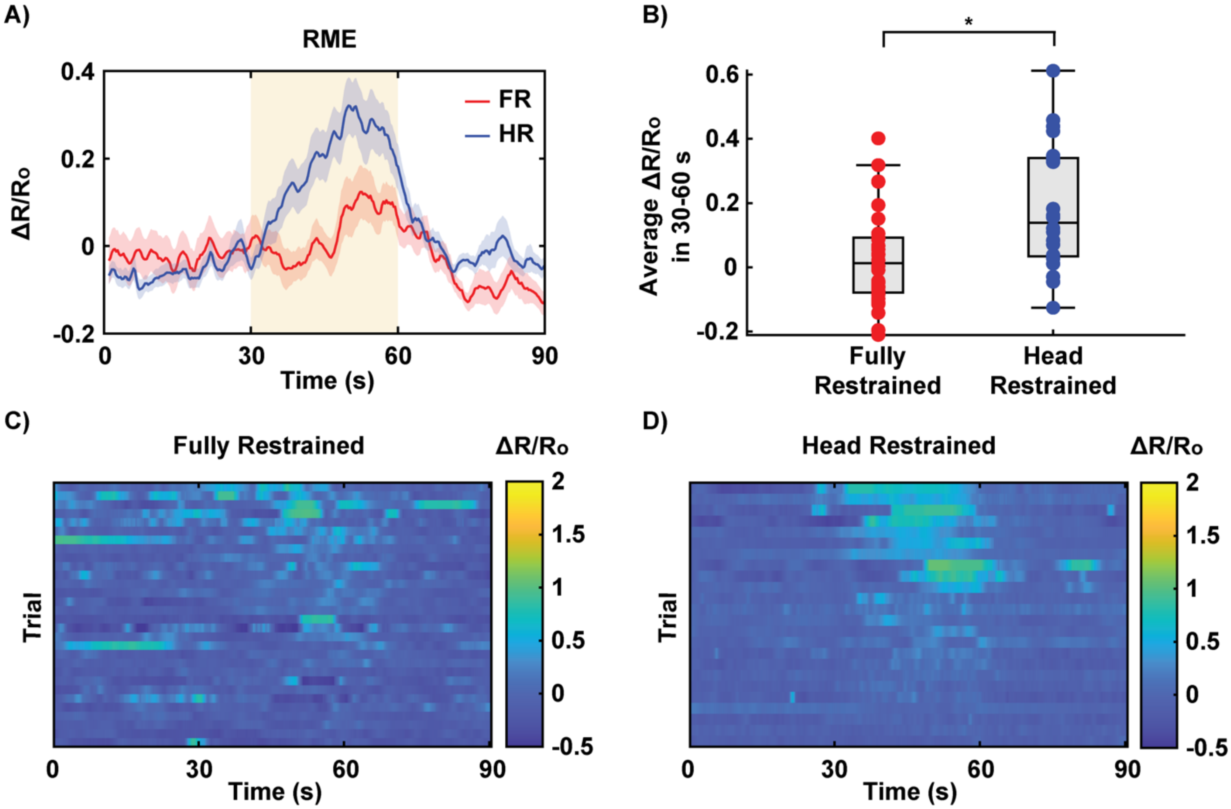


Figure S3: Difference in RME neuron response in fully-restrained vs head-fixed animals. A) Trial-averaged RME activity traces (ΔR/R0) of AVE in fully-restrained (FR) and head-fixed (HR) worms. B) Comparison of the average ΔR/R0 values between fully-restrained and head-fixed animals in the 30-60 window. Each dot represents an individual stimulation trial. The box plot displays the median along with the lower and upper quartiles. Statistical significance was determined using two-sample t-tests, with asterisks denoting significance levels: *P < 0.05, **P < 0.01, ***P < 0.001. C,D) Heatmap showing RME neuron activity (ΔR/R0) across individual trials for fully restrained and head-fixed worms. Each row represents one trial, with the stimulus applied in the 30–60 second window.

bioRxiv preprint doi: https://doi.org/10.1101/2025.11.03.686171; this version posted November 4, 2025. The copyright holder for this preprint (which was not certified by peer review) is the author/funder, who has granted bioRxiv a license to display the preprint in perpetuity. It is made available under aCC-BY 4.0 International license.

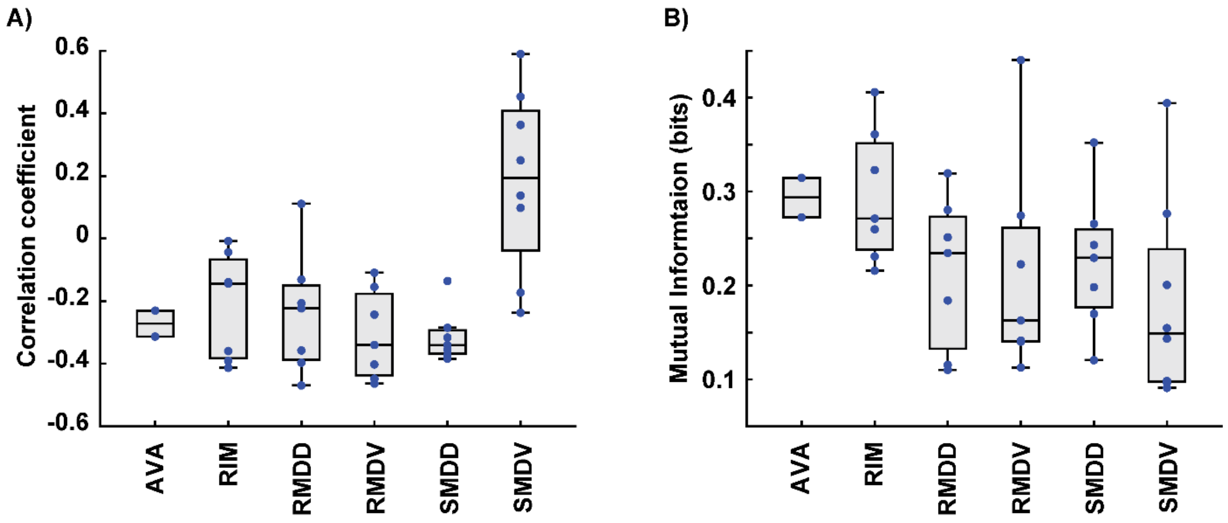


Figure S4: Correlation of neuronal activity with behavior, represented as body wave frequency and ternary classification. A) Pearson correlation coefficients representing the relationship between neuronal activity and body wave frequency for each neuron. Each dot corresponds to a neuron trace from a recording consisting of two trials. A positive correlation coefficient indicates an association with forward movement, while a negative value indicates an association with backward movement. B) Mutual Information between neuronal activity and ternary classification of behavior (forward, backward, and pause). Higher mutual information values indicate a stronger relationship between neuronal activity and the classified behavioral states, showing how much knowing the behavioral states reduces uncertainty about the neuronal activity, and vice versa. The box plots display the median and the lower and upper quartiles.
